# Mesenchymal-Stromal-Cell-Conditioned Media and Their Implication for Osteochondral Regeneration

**DOI:** 10.3390/ijms24109054

**Published:** 2023-05-21

**Authors:** Dana Ivanisova, Martin Bohac, Martina Culenova, Veronika Smolinska, Lubos Danisovic

**Affiliations:** 1 Regenmed Ltd., Medena 29, 811 01 Bratislava, Slovakia; danaivanisova@gmail.com (D.I.); bohac.md@gmail.com (M.B.); 2 Institute of Medical Biology, Genetics and Clinical Genetics, Faculty of Medicine, Comenius University, Sasinkova 4, 811 08 Bratislava, Slovakia; martina.culenova@fmed.uniba.sk (M.C.); smolinska7@uniba.sk (V.S.); 3 Centre for Tissue Engineering and Regenerative Medicine–Translational Research Unit in the Branch of Regenerative Medicine, Faculty of Medicine, Comenius University, Sasinkova 4, 811 08 Bratislava, Slovakia; 4 National Institute of Rheumatic Diseases, Nábrežie I. Krasku 4, 921 12 Piešťany, Slovakia

**Keywords:** mesenchymal stem cells, conditioned medium, secretome, paracrine signaling, osteochondral regeneration

## Abstract

Despite significant advances in biomedical research, osteochondral defects resulting from injury, an autoimmune condition, cancer, or other pathological conditions still represent a significant medical problem. Even though there are several conservative and surgical treatment approaches, in many cases, they do not bring the expected results and further permanent damage to the cartilage and bones occurs. Recently, cell-based therapies and tissue engineering have gradually become promising alternatives. They combine the use of different types of cells and biomaterials to induce regeneration processes or replace damaged osteochondral tissue. One of the main challenges of this approach before clinical translation is the large-scale in vitro expansion of cells without changing their biological properties, while the use of conditioned media which contains various bioactive molecules appears to be very important. The presented manuscript provides a review of the experiments focused on osteochondral regeneration by using conditioned media. In particular, the effect on angiogenesis, tissue healing, paracrine signaling, and enhancing the properties of advanced materials are pointed out.

## 1. Introduction

Diseases of the musculoskeletal system need to be strictly managed daily in clinical medicine. Affecting patients of all age groups, these pathologies significantly impact an individual’s health, as well as their psychical, social, and economic status [1]. Acute traumas, autoimmune diseases, or tumors heavily affect both bone and cartilage tissues. If treated unproperly, patients may be severely disabled [2]. The physiological regeneration of cartilage and bone is a complex process that involves tight cooperation between cellular and molecular agents near the affected site [3]. Continuous remodeling, as a direct response to physiological or pathological stimuli, is typical for osteochondral tissue regeneration [4]. In the case of bone regeneration, strict coordination between bone resorption provided by osteoclasts and new tissue formation arranged by osteoblasts needs to be maintained to keep the required integrity and functionality of the bone [5]. Cartilage regeneration is affected by the fact that it is an avascular and aneural tissue. Moreover, resident chondrocytes, which are responsible for the production of important extracellular matrix (ECM) components, have a very low proliferative capacity, and, therefore, regeneration is considerably slowed down and, in many cases, it is not possible to achieve a satisfactory result.

Fractures represent the most common injuries of bone tissue, and the healing process involves inflammatory, proliferative, and remodeling stages [6]. Effective blood supply to the affected area is crucial for the overall process of new bone formation and is related to the recruitment of adjacent stem cells that can differentiate into blood vessels [7]. Although the tissue’s propensity for self-repair is high, lower-quality tissue can still be formed and negatively affect patients´ life. Limited vascularization of the cartilage is one of the reasons why its intrinsic self-healing capacity is so restricted and why it is practically unable to repair [8]. Currently used surgical techniques often lead to the formation of scar tissue [9]. Novel approaches provided by tissue engineering and regenerative medicine might offer promising alternatives for the effective healing of even large defects affecting bone, as well as cartilage. Due to their differential healing capacity, mesenchymal stromal cells (MSCs) have been intensively studied concerning osteochondral regeneration [10].

**Figure 1:**
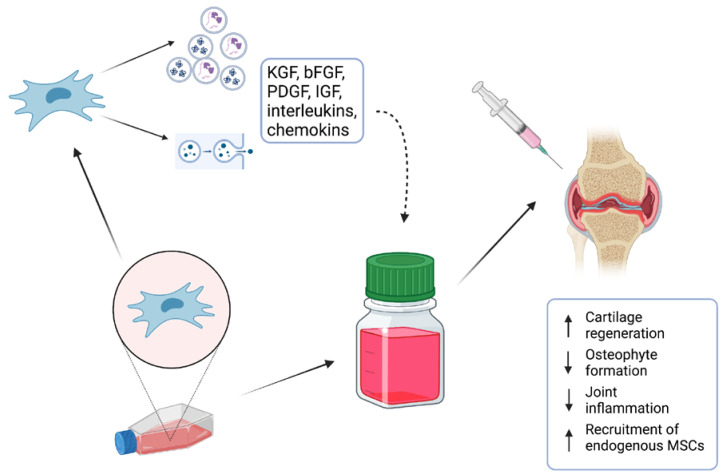
MSC-conditioned medium (MSC-CM) containing various proteins, lipids, cytokines, mRNAs, and growth factors is a potential candidate for cell-free therapy of osteochondral defects.

Many in vitro studies have investigated their potential when applied as stem-cell therapy or combined with scaffolds [11]. Long-running and detailed investigations of MSCs have helped us to elucidate complex processes which are responsible for their biological actions. It is accepted that MSCs operate via the secretion of active molecules such as proteins, lipids, cytokines, mRNAs, and growth factors, which seem to have a direct effect on tissue regeneration [12]. To minimize the risks for patients, attention is currently drawn to the evaluation of the effect of the MSC-conditioned medium (MSC-CM) as a potential candidate for cell-free therapy (Figure 1). In addition, various studies that focused on clinical MSC application have suggested that their regenerative properties are not related to the proximity of the applied cells to the targeted tissue [13]. Moreover, it was also described that the applied cells could not maintain their lifespan for a long time, and, therefore, their effect needed to be provided by secreted bioactive molecules [13]. The MSC secretome presents a package of bioactive agents secreted into extracellular space [14]. It can be easily obtained with collection of CM and various techniques and protocols have been already applied for its analysis [15].

The presented manuscript offers a comprehensive review of the experiments focused on bone or cartilage regeneration by using MSC-CM. In particular, the effect on angiogenesis, tissue healing, paracrine signaling, and enhancing the properties of advanced materials are pointed out. The literature search was conducted using the electronic database PubMed/Medline (December 2022). To obtain the most relevant primary results, the following keywords were used: conditioned medium, bone repair, cartilage repair, and tissue engineering. According to our search, MSC-CMs were mainly obtained by standardized methodologies including the establishment of the cell culture from harvested MSCs, and basic cell culturing until 80% confluency: (c) cell washing, switching from standard culture media to serum-free media; (d) the collection of MSC-CM; (e) the purification of MSC-CM via centrifugation and filtration; (d) the direct use of MSC-CM or its storage by freezing. MSC-CM was derived from both animal and human MSCs. SCs were mostly harvested from bone marrow and adipose tissue. Other sources represented dental tissue or umbilical cord. Most authors used serum-free media for conditioning. Interestingly, hypoxic culture conditions were applied only in four cases.

To make this review reader-friendly and clear, the obtained articles were logically categorized as follows: (a) the preparation of MSC-CM, (b) scaffold pretreating using MSC-CM, (c) MSC-CM-based immunomodulation, (d) the effect of MSC-CM on angiogenesis, (e) the effect of MSC-CM on bone/cartilage healing. The most important findings of the selected studies are described in the following text.

## 2. Preparation of MSC-Conditioned Medium

MSC-CM is a type of cell culture medium that has been conditioned or modified by the paracrine action of MSCs. This medium contains various growth factors, cytokines, and other molecules secreted by the MSCs and can be used for various purposes, such as promoting cell growth and differentiation, studying cell signaling pathways, and evaluating the therapeutic potential of MSCs [16]. The most common sources of stem cells which are used for MSC-CM “fabrication” are bone marrow MSCs, adipose tissue MSCs, and dental pulp MSCs [17,18].

The first step of MSC-CM production is the isolation and in vitro expansion of MSCs under static or dynamic conditions to obtain a suitable number of cells before starting the conditioning process. Recently, a fully closed, automated, and GMP-compliant cell expansion system (e.g., CliniMACS Prodigy^®^) was developed which can be used to allow the large-scale production of MSCs. After a precise characterization of MSCs, culture media are replaced by “starving” serum-free basal media. In this step, MSCs start to secrete various bioactive molecules and extracellular vesicles. In some cases, MSCs are cultured under specific conditions, such as low oxygen tension or the addition of specific growth factors, to encourage the secretion of bioactive molecules into the media. After a certain period of time, typically 24–48 h, the conditioned media are harvested and filtered to remove any cell debris. Afterward, MSC-CM can be concentrated to increase the concentration of bioactive molecules. This can be carried out using various methods, such as centrifugation or ultrafiltration. The final step is the quality control of the fabricated MSC-CM. MSC-CM is usually analyzed for the presence of specific bioactive molecules, such as growth factors and cytokines, using techniques such as ELISA or mass spectrometry [19,20].

## 3. Scaffold Pretreating Using MSC-Conditioned Medium

Within the fields of tissue engineering and regenerative medicine, cells, scaffolds, and growth factors create the main pillars for effective tissue regeneration. Various studies confirmed that the application of scaffolds could enhance bone healing when compared to only SC application [21]. Moreover, recent studies suggested that pretreatment of the scaffolds with MSC-CM might have provided even better results. Scaffold pretreating using MSC-conditioned medium refers to a technique that involves treating a scaffold material with a conditioned medium derived from MSCs before seeding cells onto the scaffold. MSCs are known to secrete various growth factors and cytokines that can stimulate cellular proliferation, migration, and differentiation. By pretreating the scaffold with MSC-CM, the scaffold can be “primed” with these growth factors and cytokines, providing a more favorable environment for seeded cells to adhere, proliferate, and differentiate [22].

Garcìa-Ruìz and colleagues applied MSC-CM from bone marrow MSCs and used it to improve the biological properties of a 3D-printed composite scaffold [23]. The results showed that cells seeded on pretreated scaffolds could attach and proliferate better on the scaffold´s surface. In addition, chondrogenic differentiation was promoted at a higher rate as well, making this approach a promising strategy used for osteochondral regeneration. A similar technique was utilized in the following studies to repair bone tissue under in vivo conditions [5,12]. Seeded scaffolds affected by MSC-CM repaired rat calvarial defects more successfully. These outcomes were also supported by in vitro results which revealed a higher expression of osteogenic genes in applied cells. In a more recent study performed by Chang and colleagues, tissue-engineered bones were fabricated with a combination of demineralized bone matrix and MSCs to treat large segmental bone defects. They also used the pretreatment of scaffolds with MSC-CM. Their results demonstrated that MSC-CM containing significant concentrations of various growth factors had a positive effect on MSC migration, proliferation, and osteogenic differentiation [24].

The improved bone-healing capacity of MCS-CM related to electrical stimuli and a 3D culture system was investigated in a study carried out by Hwang and colleagues [25]. MSCs were seeded on collagen sponges and constructs were/were not exposed to electric stimuli. Combining the mentioned culture techniques seemed to significantly improve inflammatory-mediated bone loss repair. Ogata and coworkers used atelocollagen in combination with human MSC-CM to treat bone defects. They showed an enhanced migration of endogenous osteoprogenitor cells and accelerated bone regeneration [26]. More recently, Dilogo and coworkers conducted a large animal study investigating the effect of hydroxyapatite and MSC-CM on the treatment of critical-sized bone defects. They applied MSC-CM alone or with the supplementation of BMP-2. They demonstrated the most significant effect on total callus formation in the group treated with MSC-CM and BMP-2, while the osseous area was found to be highest in the MSC-CM group [27].

## 4. MSC-CM-Based Immunomodulation

MSCs have a strong ability to affect the immune system and thus support regenerative processes. They implement it through cell–cell contact as well as via paracrine action. For this reason, even MSC-CM has immunomodulatory effects [28,29]. MSC-CM-based immunomodulation has been explored in several disease settings, including autoimmune diseases, inflammatory disorders, and tissue injury. For instance, MSC-CM was used to treat colitis by upregulating TGF-β, IL-10, and a fraction of Treg cells, and downregulating IL-17 [30]. It was also demonstrated that MSC-CM induces apoptosis in M1 macrophages. On the other hand, they inhibit M0 macrophage apoptosis [31]. Gao and colleagues showed that MSC-CM induces macrophage M2 polarization through NF-κB and STAT3 pathways [32]. In addition, a study conducted by Liu and coworkers demonstrated an induction of M2 macrophage polarization facilitated by MSC-CM via TNF-α downregulation and IL-10, Arg-1, and CD163 upregulation [33].

MSC-CM also induces the apoptosis of neutrophils [34]. Moreover, it was reported that MSC-CM increases the number of regulatory T (Treg) cells and, thus, alleviates periodontitis and supports the regeneration of periodontal bone [35]. In the present study, it was demonstrated that MSCs cultured in hypoxia or the presence of anti-inflammatory agents increases their immunomodulatory effect. Therefore, MSC-CM fabricated from cultivated MSCs with improved immunomodulatory potential could be useful for osteochondral regeneration [36,37].

## 5. Effect of MSC-CM on Angiogenesis

The neovascularization of an implanted tissue-engineered construct is a crucial point that determines the further success of tissue regeneration [38]. The failure of effective angiogenesis, mostly observed in the repair of large tissue defects, remains a point of concern in regenerative medicine [39].

The effect of the MSC secretome on angiogenesis has been evaluated in the following studies. Hoch and coworkers described significantly higher tubule formation of endothelial colony-forming cells (ECFCs) when treated with MSC-CM [40]. Moreover, the chemotactic effect of MSC-CM on ECFCs was also determined as the migration of cells was observed when cultured in a transwell system. To elucidate concrete agents in the MSC-secretome that play a crucial role in the process of angiogenesis, Nossin and his team focused on soluble factors secreted in the MSC-CM of chondrogenically differentiated bone marrow MSCs [41]. The results showed that the proangiogenic potential of the MSC secretome might have been enhanced by blocking two specific factors, Serpin E1 and Indian Hedgehog, which behaved as antiangiogenic agents. On the other hand, another study described that the activation of the Hedgehog signaling pathways together with the use of MSC-CM stimulated osteogenesis [42]. A positive effect of MSC-CM on neovascularization as well as bone healing was confirmed in another study conducted by Wang et al. [43]. The authors focused on the concentrations of the vascular endothelial growth factor (VEGF) and interleukin 6 (IL-6) in the MSC-CM derived from the human primary MSCs. Interestingly, CM was collected under hypoxic conditions. To evaluate the healing effect of the MSC secretome in vivo, collagen sponges delivering MSC-CM were applied subcutaneously or into fibular defects presented in diabetic rats. The results showed better tissue ingrowth, fracture healing, as well as neovascularization at the implanted site when compared with other experimental groups.

Evaluating the effect of oxygen levels on the quality of MSC-CM was the point of interest in the following study [44]. Human dental pulp cells (hDPCs) were cultured both under normoxic or hypoxic culture conditions and the secretome was analyzed under in vitro as well as in vivo conditions. The outcomes showed that hypoxic conditions enhanced the proangiogenic potential of MSC-CM which was related to the secretion of VEGF-A and angiopoietin-2 (Ang-2) by hDPCs. Moreover, the application of hypoxic MSC-CM together with the distraction osteogenesis procedure enhanced bone healing. High secretion levels of VEGF were also measured in MSC-CM after the cells had been treated by the specific phytomolecule icariin [45]. In a study carried out by Kim et al. [46], the authors also pointed out the high secretion levels of VEGF found in MSC-CM. MSC-CM was derived from fucoidan-induced MSCs, which rapidly differentiated into osteoblasts. In addition, the cooperation between differentiated osteoblasts and endothelial cells was elucidated. The results showed that MSC-CM from induced cells significantly promoted the phosphorylation of mitogen-activated protein kinases and the PI3K/AKT/eNOS signaling pathway, having a positive effect on the angiogenesis of the human vein endothelial cells.

Santos and colleagues investigated two types of MSC-CM and their effect on cell migration [47]. In particular, CM harvested from modified mouse bone marrow MSCs overexpressing human leukemia inhibitory factor (LIF) was compared with MSC-CM from unmodified bone marrow MSCs to assess angiogenic potential. The results showed that MSC-CM derived from modified MSCs significantly increased migration and the in vitro tube formation of endothelial cells. An interesting finding was described in a study conducted by Kohli et al. [48]. The authors selected phenotypically specific MSCs with the improved ability of osteogenesis CD271+MSCs. MSC-CM derived from these cells was compared to the secretome harvested from plastic-adherent MSCs. According to the results from in vitro angiogenic assays, CD271+MSC-CM showed less potential. This finding was also confirmed when applied in vivo. On the other hand, its beneficial effect was determined in the early healing phase of the osteochondral lesion and matrix deposition was enhanced. The authors also discussed that this might have also been the reason for the lower angiogenic capacity, as the presence of glycosaminoglycans (GAGs) might have had an inhibitory effect on neovascularization. Taken together, these outcomes suggested that CD271+MSCs might have been good candidates for osteochondral regeneration.

## 6. Effect of MSC-CM on Bone/Cartilage Healing

Healing of the hard tissues is a long process, requiring at least 21 days [49]. It depends on the recruitment and differentiation of endogenous progenitors, proper vascularization, the balance between pro- and anti-inflammatory reactions, regulation by specific pathways, etc. [50]. Various studies have shown that MSC-CM can have positive effects on bone and cartilage healing. For bone healing, MSC-CM has been shown to increase the activity and differentiation of osteoblasts, the cells responsible for bone formation. In addition, MSC-CM can also stimulate the proliferation and migration of endothelial cells, which can enhance blood vessel formation and supply nutrients to the healing bone tissue. For cartilage healing, MSC-CM has been shown to promote chondrocyte proliferation and extracellular matrix synthesis, leading to the formation of new cartilage tissue. MSC-CM also has anti-inflammatory properties, which can help reduce inflammation in the joint and promote healing. These aspects and their relation to the application of MSC-CM are described in the following text (Table 1).

Katagiri and colleagues applied human bone-marrow-derived CM (BM-CM) to assess its effect on osteogenic differentiation and potential cell recruitment [51]. After culturing animal MSCs in BM-CM, it was found that the examined secretome increased cell migration as well as osteogenic differentiation. Moreover, when applied in vivo via a collagen sponge soaked in the BM-CM, healing of the rat calvarial defect was observed. The positive differentiation effect of MSC-CM was described in a study carried out by Li et al. [52]. CM derived from osteogenically induced MSCs could potentiate osteogenic differentiation and the migration of exogenous MSCs. Another research work focused on the multilineage differentiation of dental pulp cells induced by two types of MSC-CM, which were derived from dental apical papilla SCs (SCAPs) or bone marrow SCs [53]. The results revealed that SCAPs-CM had a significantly better effect on DPCs concerning osteo/odontogenic as well as neurogenic differentiation. Migration was also enhanced. On the other hand, BM-CM provided better a angiogenic differentiation of dental pulp cells. The outcomes of the experiment performed by Pellagalli and coworkers demonstrated that the effect of MSC-CM on cell migration was related to the activation of Akt and Erk intracellular signal pathways [54]. Moreover, cells that were influenced by MSC-CM showed higher levels of two specific membrane proteins, Aquaporin 1 and C-X-C chemokine receptor type 4. In addition, one experiment suggested that the chemotactic effect of MSC-CM might have been associated with calcium ions and p38 [47].

The healing effect of the MSC secretome on animal chondrocytes was studied in the context of potential osteoarthritis management [55]. The study pointed out that MSC-CM derived from dental-pulp-derived stem cells had a positive effect on the lifespan of murine articular chondrocytes. Interestingly, its immunomodulatory effect was also established, as cytokine-induced (TNF-α, IL-1β) articular chondrocytes expressed the *TIMP-1* gene at a higher rate. Li et al. used inflammatory cytokines to stimulate MSCs and the authors subsequently evaluated this effect on osteogenesis [56]. The osteoblast-like cell line MG63 was treated with MSC-CM harvested from pretreated MSCs. High concentrations of the following cytokines were present in the secretome: IL-6, hepatocyte growth factor (HGF), VEGF, and transforming growth factor beta (TGF-β). Additionally, enhanced osteoblast differentiation and cell recruitment together with increased mineralization were observed as well.

Clarification of the molecular mechanisms responsible for MSC-CM osteogenic potential was the point of interest in a study conducted by Sun et al. [57]. The authors affected osteoblasts with MSC secretome. Cell analyses showed that within the first 6 days, an inhibition of osteoblast proliferation was present. The expression levels of osteogenic-related genes were also reduced. The researchers associated this phenomenon with the downregulation of the *Runx2* gene. Moreover, this relation was assigned to the concentration of the applied MSC-CM. However, the decreased osteogenic potential was transient and was regained after 7 days of culture.

The secretome derived from adipose-tissue-derived stem cells was thoroughly analyzed in the following study [58]. Multiple trophic factors stimulating osteoblast as well as osteoclast biological activity were described, among them periostin, fibronectin, beta-2 microglobulin, etc. Furthermore, the signaling pathways responsible for osteoblast/osteoclast differentiation under the influence of adipose tissue MSC-CM were revealed as well. The results showed that the MSC-CM stimulated osteoblast reproduction and differentiation via ERK/JNK activation and induction of osteoclast division and differentiation by ERK/JNK/p38 activation under in vitro conditions. Its paracrine effect on the healing of surgical bone lesions was investigated by Linero et al. [59]. When applied in vivo, the impact of MSC-CM on bone healing was comparable to adipose-tissue-derived stem cell administration, highlighting the potential of MSC-CM to be used as cell-free therapy. Interestingly, the authors also compared the quality of the secretome obtained under normoxic/hypoxic conditions. It was established that hypoxic conditions enhanced the higher secretion of the bioactive factors into MSC-CM. Out of 43 secreted molecules, 11 were identified as significant for bone regeneration with the highest concentrations detected for insulin growth factor 1 (IGF-1), IL-6, and monocyte chemoattractant protein-1 (MCP-1). In the following study, mechanical stimuli together with the MSC secretome were applied to support bone regeneration [60]. The MSC-CM was harvested from bone-marrow-derived MSCs, which were cultured under a cyclic stretch stimulation. Subsequently, the osteogenic and angiogenic potential of the obtained secretome were analyzed. In addition, CM was also applied in vivo to restore the rat calvarial defect. The results showed that mechanical stimuli upregulated the expression of osteogenic- and angiogenic-related genes, especially BMP-2, BMP-4, VEGF-A, and PGDF-AA, when compared to the unstimulated control group. When applied in vivo, MSC-CM from stimulated cells promoted better bone healing. Saiz Jr. et al. [61] described a better osteogenic effect of secretome derived from MSCs which were cultured as 3D forms (spheroids) under hypoxic conditions when compared to a standard monolayer culture system. Moreover, the synergistic effect of MSC-CM and myokines in the context of osteogenesis and bone healing was described as well.

Santos and his team described the critical point of view on the positive osteogenic effect of MSC-CM [47]. The authors influenced osteoblasts with MSCs indirectly via a coculture system or MSC-CM derived from rat bone-marrow-derived stem cells under osteogenic conditions. The results showed (in both cases) that osteoblast proliferation, ALP activity, osteogenic gene expression, and extracellular matrix mineralization were inhibited. The authors pointed out that the simultaneous application of MSCs together with osteoblasts could not bring the expected therapeutic effect for the patients. The trophic effect and influence of different types of MSCs (adipose/bone marrow) on chondrogenesis were investigated in a study carried out by Pleumeekers et al. [62]. Stem cells were mixed with articular chondrocytes and studied under in vitro and in vivo conditions. Cellular interactions between SCs and chondrocytes were examined via multiple approaches to cell culturing. When MSC-CM was applied, enhanced chondrogenesis and extracellular matrix formation were observed. The authors confirmed positive trophic effects on chondrocytes. Moreover, its antiapoptotic effect was described as well [63,64]. These outcomes indicated MSC-CM as a promising candidate for cartilage repair.

**Table 1:** Overview of studies which investigated the effect of MSC-CM on osteochondral regeneration.

Cell Type Used for MSC-CM Preparation	Study Design	Effect	Ref.
Human bone marrow MSCs	MSC-CM pretreatment of rat bone marrow MSCs + collagen sponge application into rat calvarial bone defects	↑ migration ↑ proliferation ↑ expression of osteogenic marker genes	[46]
Murine bone marrow MSCs	Murine bone marrow MSCs + osteogenic factors; evaluation of MCS-CM on exogenous MSCs	↑ migration ↑ expression of osteogenic marker genes	[47]
Human dental apical papilla stem cells and bone marrow stem cells	Evaluation of MSC-CM’s effect on dental pulp stem cells (in vitro)	↑ migration ↑ osteo/odontogenic differentiation	[48]
Ovine bone marrow MSCs	Evaluation of MSC-CM on ovine bone marrow MSCs (in vitro)	↑ migration activation of Akt and Erk pathways	[49]
Human dental pulp stem cells	Evaluation of MSC-CM on immature murine articular chondrocytes (in vitro)	↑ chondrocytes lifespan ↑ *TIMP-1* gene expression (immunomodulatory effects)	[50]
Human adipose tissue MSCs	Evaluation of MSC-CM on osteoblasts and osteoclasts (in vitro)	↑ proliferation ↑ differentiation	[54]
Human adipose tissue MSCs	MSC-CM + human blood plasma hydrogels; application into male New Zealand white rabbits	↑ bone regeneration	[55]
Human bone marrow MSCs	MSC-CM + hypoxia + 3D culture; evaluation of the effect on bone regeneration (in vitro)	↑ osteogenic response	[56]
Human adipose tissue MSCs	MSC-CM + chondrocytes; evaluation of MSC-CM on chondrogenesis (in vitro)	↑ cartilage matrix production	[57]
Human umbilical cord MSCs	MSC-CM; evaluation of the effect on chondrocytes (in vitro)	↑ antiapoptotic effect	[58]
Human adipose tissue MSCs	MSC-CM, intra-articular injection into rat osteoarthritic knee, and evaluation of the effect on cartilage regeneration	↑ expression of chondrogenic marker genes ↓ inflammatory cytokines ↓ inflammation-induced BMPs	[61]

The therapeutic effect of MSC secretome on tendon–bone healing was confirmed in vivo in a study carried out by Chen et al. [65]. The authors contributed this finding to the fact that MSC-CM derived from human bone-marrow-derived stem cells promoted macrophage polarization into the M2 (anti-inflammatory) phenotype. A molecular mechanism was also revealed as this phenomenon was linked to the Smad2/3 signaling pathway.

The intra-articular application of MSC-CM to manage osteoarthritis was performed in the following study [66]. In addition, the dose-dependent effect of the secretome derived from adipose-tissue-derived SCs was also compared. The best results were observed when high doses of CM were administered to the affected joint (rat knee). It was reflected in a significant decrease in inflammatory cytokines and inflammation-induced BMP, which were responsible for the imbalance of the cartilage homeostasis (catabolic versus anabolic reactions). Along with these results, gene expression related to cartilage repair (collagen II, SOX9, and SRY-box transcription factor 9) was significantly higher when compared to the control group.

The modulatory and anti-inflammatory effects of MSC-CM were also the point of interest of the experiments performed by Lee et al. [67]. The secretome derived from different types of MSCs was analyzed under both physiological and inflammatory conditions. More than 90 proteins were commonly found in all types of MSC-CM harvested under inflammatory conditions. The authors also described the most relevant ones associated with cartilage repair in the pathological environment, whilst remarkably high concentrations were detected for TSG-6 and thrombospondin-1. The results showed that the secretion of paracrine factors related to cartilage repair was induced by inflammatory conditions.

Overall, MSC-CM has the potential to be a promising therapeutic agent for bone and cartilage healing. However, further research is needed to fully understand its mechanisms of action and optimize its clinical applications.

## 7. Conclusions

Recently, various MSC-CMs have been produced to improve the regeneration of osteochondral defects. Their application mostly showed promising results, both in vitro and in vivo. The main effect of MSC-CM is associated with its unique composition. Different bioactive molecules and extracellular vesicles may support cell proliferation, viability, migration, and differentiation into chondrogenic and osteogenic lineages. They also positively influence the production of proteins typical for cartilage and bone ECM and thus contribute to ECM remodeling, a crucial process for osteochondral regeneration. They have a significant anti-inflammatory effect and proangiogenic effect, as well. When compared with cell-based therapies, the major advantage of using MSC-CM is that it avoids the need for the direct transplantation of the cells themselves, which can be challenging due to issues such as immunogenicity and limited cell survival. Instead, the bioactive molecules in the CM can be delivered directly to the target tissue, which can result in similar therapeutic effects. Moreover, the utilization of MSC-CM can overlap the biosafety problems associated with the risk of tumor formation.

However, it will be necessary to carry out further research before the translation of MSC-CM to human medicine. Some issues will need to be resolved, such as the exact dosage of MSC-CM, the way of administration, and the effect of MSCs from different tissues, and long-term biosafety studies and proof-of-concept studies evaluating the therapeutic effects of MSC-CM in large animal disease models should be carried out. Last but not least, great attention must be paid to the development, comprehensive standardization, and validation of CM-MSC production protocols, mainly in clinical grade.

## Data Availability

Not applicable.
